# Modulation of Monocyte-Driven Myositis in Alphavirus Infection Reveals a Role for CX_3_CR1^+^ Macrophages in Tissue Repair

**DOI:** 10.1128/mBio.03353-19

**Published:** 2020-03-03

**Authors:** Ali Zaid, Kothila Tharmarajah, Helen Mostafavi, Joseph R. Freitas, Kuo-Ching Sheng, Suan-Sin Foo, Weiqiang Chen, Jelena Vider, Xiang Liu, Nicholas P. West, Lara J. Herrero, Adam Taylor, Laura K. Mackay, Daniel R. Getts, Nicholas J. C. King, Suresh Mahalingam

**Affiliations:** a Menzies Health Institute Queensland, Griffith University, Southport, Queensland, Australia; b School of Medical Science, Griffith University, Southport, Queensland, Australia; c Institute for Glycomics, Griffith University, Southport, Queensland, Australia; d The Doherty Institute for Infection & Immunity, Department of Microbiology and Immunology, The University of Melbourne, Parkville, Victoria, Australia; e The Australian Research Council Centre of Excellence in Advanced Molecular Imaging, The University of Melbourne, Parkville, Victoria, Australia; f The Discipline of Pathology, School of Medical Sciences, Sydney Medical School, The University of Sydney, Sydney, New South Wales, Australia; g Bosch Institute, School of Medical Sciences, Sydney Medical School, The University of Sydney, Sydney, New South Wales, Australia; h Department of Microbiology–Immunology, Feinberg School of Medicine, Northwestern University, Chicago, Illinois, USA; i Interdepartmental Immunobiology Center, Feinberg School of Medicine, Northwestern University, Chicago, Illinois, USA; j Myeloid Therapeutics Inc., Cambridge, Massachusetts, USA; k Marie Bashir Institute for Infectious Diseases and Biosecurity, Sydney Medical School, The University of Sydney, Sydney, New South Wales, Australia; l Australian Institute for Nanoscale Science and Technology, The University of Sydney, Sydney, New South Wales, Australia; University of Colorado School of Medicine

**Keywords:** inflammation, macrophages, microparticles, myositis, Ross River virus, tissue repair, viral infection

## Abstract

Arthritogenic alphaviruses such as Ross River and Chikungunya viruses cause debilitating muscle and joint pain and pose significant challenges in the light of recent outbreaks. How host immune responses are orchestrated after alphaviral infections and lead to musculoskeletal inflammation remains poorly understood. Here, we show that myositis induced by Ross River virus (RRV) infection is driven by CD11b^hi^ Ly6C^hi^ inflammatory monocytes and followed by the establishment of a CD11b^hi^ Ly6C^lo^ CX_3_CR1^+^ macrophage population in the muscle upon recovery. Selective modulation of CD11b^hi^ Ly6C^hi^ monocyte migration to infected muscle using immune-modifying microparticles (IMP) reduced disease score, tissue damage, and inflammation and promoted the accumulation of CX_3_CR1^+^ macrophages, enhancing recovery and resolution. Here, we detail the role of immune pathology, describing a poorly characterized muscle macrophage subset as part of the dynamics of alphavirus-induced myositis and tissue recovery and identify IMP as an effective immunomodulatory approach. Given the lack of specific treatments available for alphavirus-induced pathologies, this study highlights a therapeutic potential for simple immune modulation by IMP in infected individuals in the event of large alphavirus outbreaks.

## Abstract (precis)

Arthritogenic alphaviruses cause debilitating inflammatory disease, and current therapies are restricted to palliative approaches. Here, we show that following monocyte-driven muscle inflammation, tissue recovery is associated with the accumulation of CX_3_CR1^+^ macrophages in the muscle. Modulating inflammatory monocyte infiltration using immune-modifying microparticles (IMP) reduced tissue damage and inflammation and enhanced the formation of tissue repair-associated CX_3_CR1^+^ macrophages in the muscle. This shows that modulating key effectors of viral inflammation using microparticles can alter the outcome of disease by facilitating the accumulation of macrophage subsets associated with tissue repair.

## INTRODUCTION

Arthritogenic alphaviruses, including Ross River virus (RRV) and Chikungunya virus (CHIKV), are mosquito-borne viruses that cause severe inflammatory musculoskeletal illnesses (1). RRV is endemic in various regions of Australia, and its distribution is broadening (2, 3), while CHIKV is distributed globally and causes recurrent pandemics involving millions of people (4–6). These alphavirus infections are associated with acute myositis and musculoskeletal tissue inflammation (e.g., joints, muscle, and bone) during systemic spread in the host, resulting in debilitating symptoms, including myalgia and arthralgia (7–9). In most cases, excessive leukocyte infiltration into the infected tissue results in significant damage, which in turn may facilitate locally amplified viral replication: it is therefore crucial to understand the mechanisms by which effector leukocyte populations infiltrate host tissue during viral infections to limit tissue damage and enhance pathogen clearance.

Animal models of arthritogenic alphavirus disease have shown that myeloid cells of the monocytic lineage are key drivers of muscle and joint synovium inflammation, and studies where monocytes/macrophages were depleted in RRV-infected mice showed a reduction in muscle inflammation and overall disease clinical score (10), although this could be conducive to poorer viral clearance (11). Monocytes, macrophages, and their associated chemotactic proteins dominate infiltrates in synovial effusions from RRV patients, though little is known about their role in muscle inflammation and damage (12–14). Recent studies have added valuable information to the field, and CD11b^hi^ Ly6C^hi^ inflammatory monocytes (IM) have been implicated in a range of inflammatory mechanisms (15, 16), and infections with arboviruses such as West Nile virus (WNV) have been shown to cause inflammation driven by IM recruited to the brain via CCL2 secreted by infected neurons.

Macrophages (MP) can be loosely distinguished as classically (M1) or alternatively (M2) activated monocytes/macrophages (17–19). M1 macrophages are characterized by elevated production of proinflammatory cytokines such as interleukin 12 (IL-12), tumor necrosis factor (TNF), and inducible nitric oxide synthase (iNOS), while M2 (tissue repair; patrolling) macrophages can be defined by the expression of Arg-1, Fizz-1/RELMα, Ym-1/Chi3l3. In addition, expression of CX_3_CR1, the fractalkine (CX_3_CL1) receptor on monocytes/macrophages has been associated with tissue repair mechanisms following injury—via an M2-like tissue repair microenvironment—though the role of the CX_3_CR1-CX_3_CL1 axis in regulating tissue repair *in vivo* is not well understood.

Studies have shown that selective inhibition of CC chemokine ligand 2 (CCL2), the main CC chemokine receptor 2 (CCR2) ligand, resulted in a significant reduction in muscle damage, lower viral titers, and reduced mononuclear cell infiltration (20–22). Conditional ablation of CCR2-expressing IM during acute RRV infection was shown to be detrimental to viral clearance (11), but depletion of IM using immune-modifying particles (IMP) abrogated infiltration of CD11b^hi^ Ly6C^hi^ IM into the brain in a model of WNV encephalitis, thus reducing pathological manifestations and tissue damage (23). Here, we explore the cellular dynamics of acute inflammation and resolution in a mouse model of RRV-induced myositis and show the relative contributions of CD11b^hi^ Ly6C^hi^ IM and CX_3_CR1^+^ CD11b^hi^ Ly6C^lo^ MP in acute inflammation and resolution, respectively, and show how modulating IM infiltration improved disease outcome by promoting a tissue repair-associated MP subset. Taken together, our data identify a new role for a muscle tissue macrophage subset and highlight the therapeutic potential of immunomodulatory microparticles in dampening inflammatory cellular responses in acute alphavirus-induced muscle inflammation.

## RESULTS

### Acute RRV-induced myositis is followed by recovery and tissue repair.

**FIG 1:**
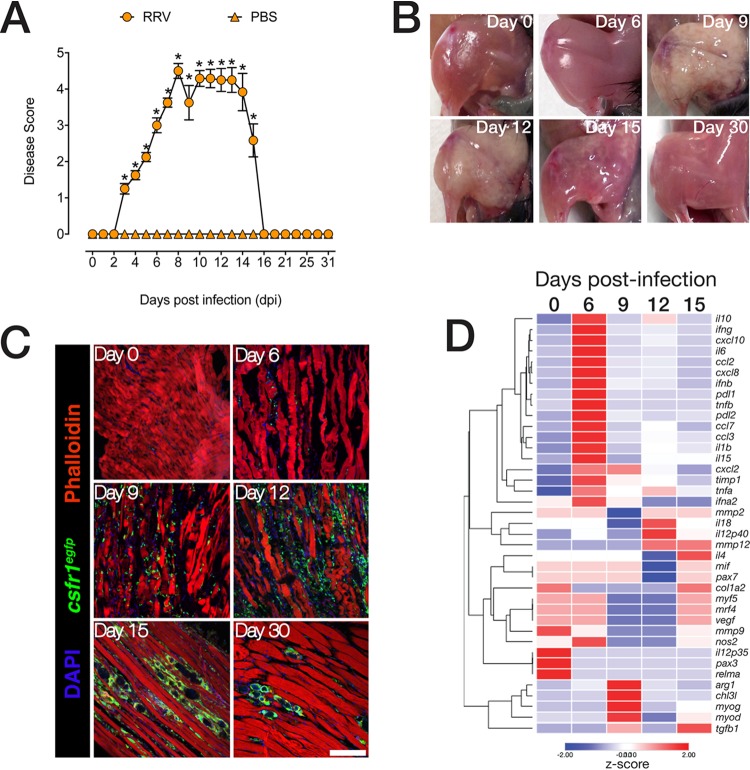
RRV-induced, mononuclear phagocyte-driven myositis leads to severe muscle damage followed by muscle tissue recovery. (A) RRV disease score in 21-day-old C57BL/6 mice infected with RRV T48 (10^4^ PFU s.c.) or mock infected with PBS. Mice were monitored daily for signs of musculoskeletal dysfunction and loss of hind limb function. Data are means ± standard errors of the means (SEM) (error bars) (five mice per group) from three independent experiments. Values that are significantly different (*P* < 0.05) by one-way ANOVA and Dunnett’s posttest are indicated by an asterisk. (B) Macrographs of quadriceps muscles showing tissue damage and fibrosis following RRV infection, followed by disease recovery and resolution. The images (four to six mice per group) are representative of three independent experiments. (C) Immunofluorescence staining of quadriceps muscle of C57BL/6 *csf1r^eGFP^* mice (*n *= 4 to 6 per group) infected with RRV. Quadriceps muscles were collected at 0, 6, 9, 12, 15, and 30 dpi. Sections (14 μm) were stained with 4′,6-diamidino-2-phenylindol (DAPI) and phalloidin. The images are representative of three independent experiments. Bar  = 50 μm. (D) Heatmap of unsupervised hierarchical clustering of gene expression analysis in the muscle tissue of RRV-infected mice. Quadriceps muscle was collected 0, 6, 9, 12, and 15 dpi and processed for quantitative real-time PCR. Data are normalized to hypoxanthine phosphoribosyltransferase (HPRT) and differential gene expression of shown as a *z*-score. Data (means ± SEM; *n* = 5 mice per group) are from two independent experiments.

To assess the kinetics of muscle tissue inflammation and repair following RRV infection, C57BL/6 (wild-type [WT]) mice were infected subcutaneously with 10^4^ PFU of the mouse virulent RRV T48 strain as described previously (24, 25). Mice were scored according to clinical manifestations from the onset of hind limb dysfunction at 6 or 7 days postinfection (dpi), to the acute phase at 9 or 10 dpi with severe hind limb dysfunction, lethargy, and muscle tissue damage (Fig. 1A). During the acute phase, mice displayed moderate-to-severe motor impairment, were unable to walk or stand on their hind legs, and often dragged their hind legs when moving. From 13 to 15 dpi, mice regained hind limb function and progressed toward full recovery, approximately around 15 to 16 dpi. Macroscopic observations of hind leg muscles shows that tissue integrity was severely compromised during the acute phase of the disease (Fig. 1B), with myofiber repair apparent from 15 dpi and followed by complete recovery by 30 dpi.

To understand the relationship between muscle damage and inflammatory dynamics, we infected *csf1r^eGFP^* mice (eGFP stands for enhanced green fluorescent protein) with RRV and performed immunofluorescence staining of quadriceps tissue sections (Fig. 1C). *csf1r^eGFP^* mice express GFP under the control of the *csf1r* gene, which encodes the macrophage colony-stimulating factor (M-CSF) receptor, resulting in constitutive GFP expression in all mononuclear phagocytic cells (26). Phalloidin stain showed a loss of myofiber integrity during acute inflammation at 9 to 12 dpi, concomitant with a substantial infiltration of GFP-positive (GFP^+^) cells. This was followed by recovery of muscle tissue integrity and clearance of inflammatory infiltrates (30 dpi). Next, we characterized gene expression kinetics in the muscle over the course of RRV disease by quantitative real-time PCR (qRT-PCR). Expression of proinflammatory chemokine (*Ccl2*, *Ccl3*, *Ccl7*, *Cxcl10*, and *Cxcl2*) and cytokine (*Il6*, *Il1b*, *Tnfa*, *Tnfb*, and *Il15*) mRNA was highest at 6 dpi. Expression of myogenic transcription factors *Myod1*, and myogenin (*Myog*) were highest at 9 dpi, while the expression of myogenic factor 6 (*Mrf4/Myf6*), myogenic factor 5 (*Myf5*), as well as collagen type 1α2 (*Col1a2*) and paired box 3 (*Pax3*) and *Pax7*, associated with tissue regeneration after injury was highest at 15 dpi (Fig. 1D). Interestingly, mRNA expression of genes associated with tissue repair such as *Tgfb*, *Mmp9*, and *Mmp12* were sustained by 15 dpi.

### Acute inflammatory CD11b^hi^ Ly6C^hi^ monocyte infiltration is followed by the establishment of CD11b^hi^ Ly6C^lo^ CX_3_CR1^+^ macrophages during resolution.

**FIG 2:**
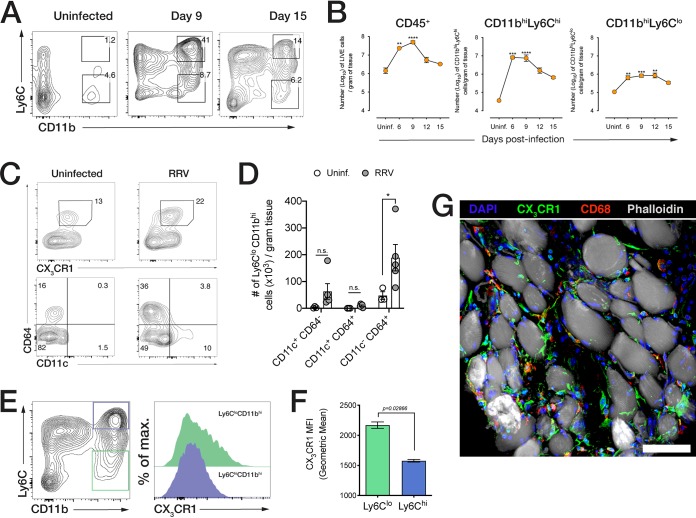
RRV-induced acute myositis is dominated by inflammatory monocytes, and recovery is associated with the accumulation of CX_3_CR1^+^ CD11b^hi^ Ly6C^lo^ cells. (A) Representative flow cytometry plots (from at least three independent experiments) of the cells that infiltrated the muscle of uninfected mice and RRV-infected mice at 9 dpi and 15 dpi. Cells were gated from LIVE/DEAD^−^ CD45^+^ populations. Parent population frequency is shown above the gates. (B) Quantification of total CD45^+^ cells, CD11b^hi^ Ly6C^hi^ inflammatory monocytes, and Ly6C^lo^ CD11b^hi^ cells in quadriceps muscle at 6, 9, 12, and 15 dpi. Data (means ± SEM; *n* = 4 to 6 mice per group) are from three independent experiments. Values that are significantly different by one-way ANOVA (Kruskal-Wallis test) with a Dunn’s multiple-comparison test are indicated by asterisks as follows: **, *P* < 0.05; ***, *P* < 0.0005; ****, *P* < 0.0001. Uninf., uninfected. (C) CX_3_CR1 and CD11c expression in CD64^+^ CD11b^hi^ Ly6C^lo^ cells in the quadriceps muscle of uninfected and RRV-infected mice at 12 dpi. The bottom panel (CD11c^+^ CD64^+^ gates) is derived from the CD64^+^ CX_3_CR1^+^ gates (top panel). The parent population frequency is shown. Data are representative of two independent experiments (*n* = 5 mice per group). (D) Quantification of CD11c- and CD64-expressing populations in CX_3_CR1^+^ CD11b^hi^ Ly6C^lo^ macrophages in the muscle of RRV-infected and PBS-inoculated mice at 12 dpi. Data (means ± SEM; *n* = 5 mice per group) are representative of two independent experiments. *, *P* < 0.05 by Mann-Whitney U test. (E) Offset histogram of CX_3_CR1 expression in CD11b^hi^ Ly6C^lo^ and CD11b^hi^ Ly6C^hi^ cells in quadriceps of mock-infected or RRV-infected mice at 15 dpi. max., maximum. (F) Geometric mean fluorescence intensity (MFI) of CX_3_CR1 expression in CD11b^hi^ Ly6C^lo^ and CD11b^hi^ Ly6C^hi^ cells at 15 dpi. Data (means ± SEM; *n* = 4 mice per group) are from three independent experiments. The *P* value shown was determined by the Mann-Whitney U test. (G) Confocal microscopy of quadriceps muscle cryosections from RRV-infected mice at 15 dpi. Sections were stained with Hoechst 33258 (nuclei), anti-CX_3_CR1 antibody, anti-CD68 antibody, and phalloidin. Bar = 30 μm.

The relative contributions of different monocyte/macrophage subsets and the dynamics of resolution of inflammation during the recovery phase are poorly understood. WT mice were infected with RRV and quadriceps muscles harvested at 6, 9, 12, and 15 dpi to characterize muscle-infiltrating cell subsets using flow cytometry (Fig. 2A). Overall, CD45^+^ leukocytes are most abundant during the acute phase (9 dpi) but decline as mice recover from acute inflammation at 15 dpi (Fig. 2B). Ly6C^hi^ CD11b^hi^ inflammatory monocytes constitute the main infiltrating subset, infiltrating the muscle tissue with a sharp influx at 6 dpi, which is maintained until 9 dpi, thereafter declining progressively by 15 dpi. Ly6C^lo^ CD11b^hi^ macrophages (MP), which are likely differentiated from infiltrating Ly6C^hi^ monocytes, increased moderately in number by 6 dpi and were maintained until 12 dpi, with a small subsequent decrease by 15 dpi (Fig. 2B). To understand how monocyte responses impact muscle tissue repair, we characterized the Ly6C^lo^ CD11b^hi^ subset during resolution. In the early phase of the recovery process (12 dpi), a substantial proportion of Ly6C^lo^ cells expressed CD64, a macrophage marker, as well as CX_3_CR1, the fractalkine/CX_3_CL1 chemokine receptor (Fig. 2C), and most—but not all—of these CD64^+^ CX_3_CR1^+^ cells were negative for dendritic cell marker CD11c (Fig. 2C and D). At 15 dpi, when mice show near complete recovery from RRV disease, we found that Ly6C^lo^ CD11b^hi^ cells (green offset histogram) express higher levels of CX_3_CR1 than CD11b^hi^ Ly6C^hi^ IM (blue offset histogram) (Fig. 2E and F), and immunostained muscle tissue revealed that CX_3_CR1^+^ MP were localized between muscle fibers, were distinct from CD68^+^ muscle tissue-resident macrophages, and displayed an elongated morphology in the myofiber interstitium (Fig. 2G).

### CX_3_CR1^+^ macrophages are important for tissue repair in RRV-induced myositis.

**FIG 3:**
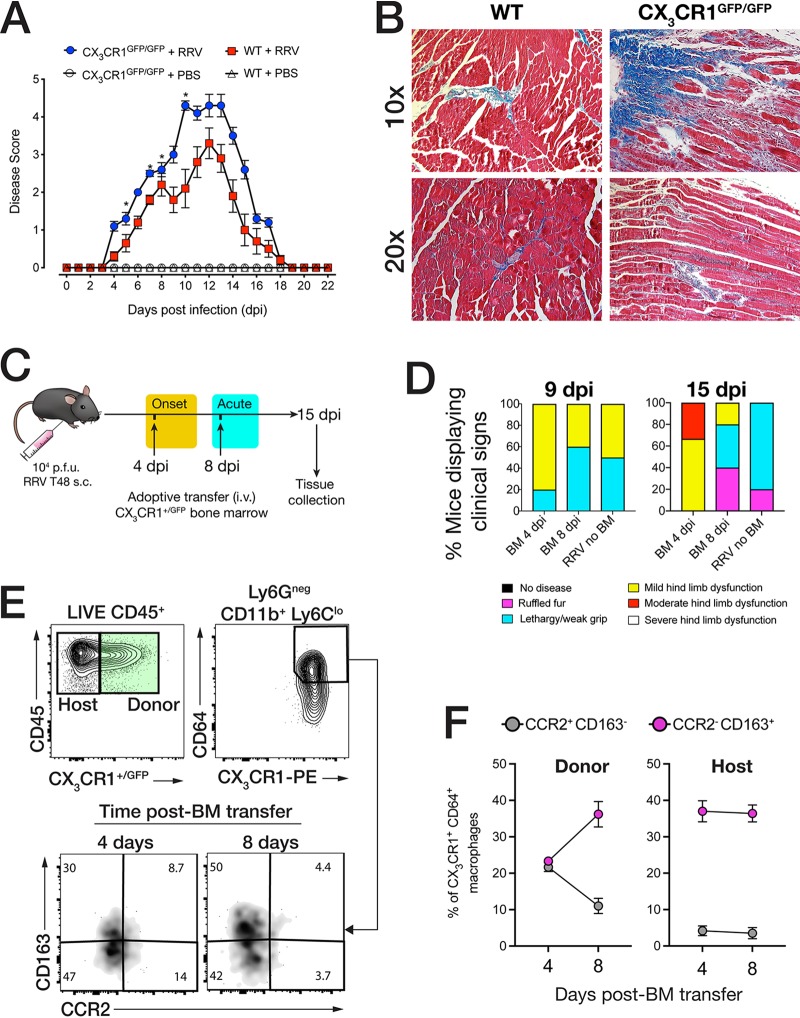
CX_3_CR1 is important for muscle recovery and the establishment of tissue repair-associated macrophages. (A) RRV disease score in 21-day-old C57BL/6 and CX_3_CR1^GFP/GFP^ (CX_3_CR1-deficient) mice infected with RRV T48 (10^4^ PFU s.c.) or mock infected with PBS. Mice were monitored daily for signs of musculoskeletal dysfunction and loss of hind limb function. Data (means ± SEM; *n* = 5 mice per group) are from three independent experiments. *, *P* < 0.05 by one-way ANOVA and Dunnett’s posttest. (B) Masson trichrome histochemical staining of paraffin-embedded quadriceps muscle sections from C57BL/6 and CX_3_CR1^GFP/GFP^ mice infected with RRV T48 (10^4^ PFU s.c.). The images (*n* = 4 or 5 mice per group) are representative of three independent experiments. (C) Schematic description of adoptive transfer of 1 × 10^5^ CX_3_CR1^+/GFP^ (CX_3_CR1 reporter) bone marrow (BM) cells to RRV-infected C57BL/6 mice at 4 dpi and 8 dpi. Quadriceps muscle was collected at 15 dpi. (D) Proportion of mice displaying clinical signs of RRV disease at 9 dpi and 15 dpi after adoptive transfer of CX_3_CR1^+/GFP^ BM cells at 4 and 8 dpi and with no BM transfer (no BM). Data (*n* = 5 in 4 dpi and 8 dpi BM groups; *n* = 6 in RRV no BM group) are representative of two independent experiments. (E) Flow cytometry analysis of donor CX_3_CR1^+/GFP^ BM cells recovered in the muscle tissue of RRV-infected C57BL/6 recipient mice following adoptive transfer at 4 dpi and 8 dpi. Representative gates show primary gating for GFP^+^ (donor BM) cells and CD64^+^ CX_3_CR1^+^ cells, from which CD163 and CCR2 gates were derived. The percentage of the parent population is shown in quadrant insets and representative of two independent experiments. (F) Percentage of CD163^+^ CCR2^−^ and CD163^−^ CCR2^+^ within the CD64^+^ CX_3_CR1^+^ donor (*CX_3_CR1^+/GFP^* BM) and host (C57BL/6) populations isolated from the muscle of RRV-infected C57BL/6 recipient mice following adoptive transfer of CX_3_CR1^+/GFP^ BM at 4 dpi and 8 dpi. Data (*n* = 5 mice per group) are representative of two independent experiments.

The establishment of CX_3_CR1^+^ MP in the muscle following acute injury suggests that this subset may be important during resolution of inflammation. To test this hypothesis, we infected CX_3_CR1-deficient mice (GFP double knock-in, CX_3_CR1^GFP/GFP^) with RRV and monitored disease progression. Compared to WT mice, CX_3_CR1^GFP/GFP^ mice displayed more severe disease (Fig. 3A), and by 15 dpi, displayed more muscle tissue fibrosis as shown by Masson trichrome stain (Fig. 3B).

Monocyte recruitment and migration to tissues occur in successive waves during acute inflammatory injury (27), and CX_3_CR1 can be expressed by both patrolling monocytes and tissue macrophages associated with resolution of inflammation (28, 29). To understand the dynamics of CX_3_CR1^+^ MP recruitment and accumulation to the muscle in RRV-induced myositis, we infected WT mice with RRV and adoptively transferred 1 × 10^5^ bone marrow (BM) cells (intravenously [i.v.]) from CX_3_CR1^+/GFP^ fluorescent reporter mice during the early onset (4 dpi) or the acute (8 dpi) phase of RRV disease (Fig. 3C). At 15 dpi, muscle tissue was collected to determine the phenotype of donor CX_3_CR1^+/GFP^ cells that migrated to the muscle. First, the cohort that had received CX_3_CR1^+/GFP^ BM at 4 dpi showed a higher proportion (80%) of mice with mild hind limb dysfunction at 9 dpi compared to the cohort that received BM at 8 dpi (40%) and those that received no CX_3_CR1^+/GFP^ BM (50%). Second, mice that had received CX_3_CR1^+/GFP^ BM at 4 dpi still displayed more advanced signs of hind limb dysfunction by 15 dpi compared to mice that received CX_3_CR1^+/GFP^ BM at 8 dpi, indicating that the timing of recruitment of CX_3_CR1^+^ cells from the circulation system to the site of acute inflammation may shape the outcome of resolution (Fig. 3D). We then analyzed the CX_3_CR1^+/GFP^ BM donor cells recovered from the muscle tissue of RRV-infected mice that received CX_3_CR1^+/GFP^ at 4 and 8 dpi by flow cytometry and found a CD64^+^ Ly6C^lo^ donor (CX_3_CR1^+/GFP^) population at 15 dpi. Notably, within these donor CX_3_CR1^+^ MP, a subset expressed CD163, a marker for alternatively activated (or M2-like) macrophages (Fig. 3E), and low levels of CCR2, a receptor for CCL2 (30). Interestingly, BM cells transferred at 8 dpi comprised a higher proportion of CD163^+^ CCR2^−^ MP compared to those transferred at 4 dpi, while the expression of CD163 and CCR2 in host CD64^+^ CX_3_CR1^+^ muscle MP remained unchanged (Fig. 3F).

### Modulation of inflammatory monocyte infiltration using microparticles ameliorates disease and promotes accumulation of muscle CX_3_CR1^+^ macrophages.

**FIG 4:**
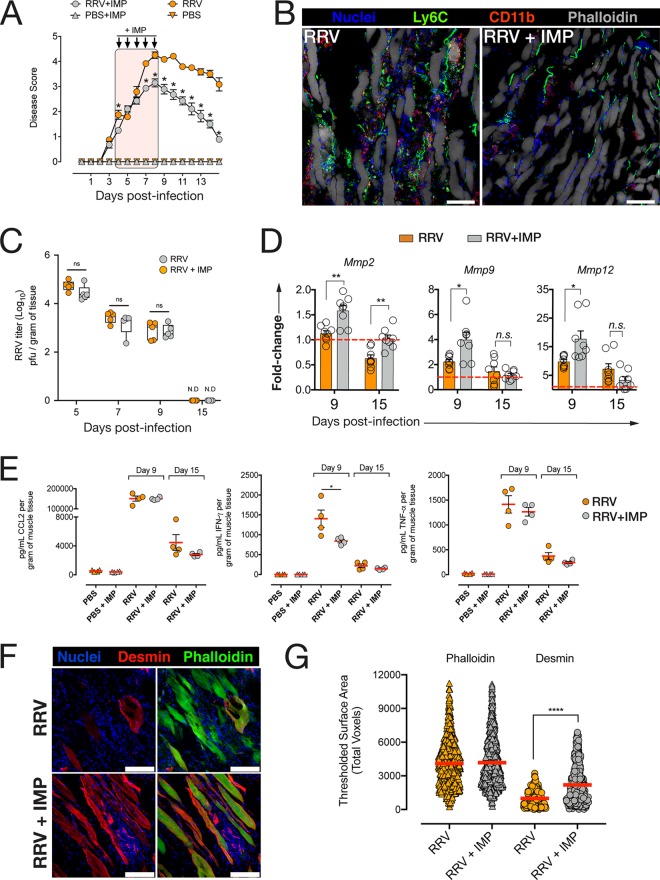
Treatment of RRV-infected mice with immune-modulating microparticles (IMP) ameliorates RRV disease and reduces quadriceps muscle damage. (A) Clinical score monitoring of RRV-infected mice treated with IMP (15 mg/kg i.p.) or vehicle (PBS) starting at 4 dpi and daily until 8 dpi. *, *P* < 0.05 by one-way ANOVA with Holm-Sidak correction. Data (means ± SEM; *n* = 5 mice per group) are from three independent experiments. (B) Confocal microscopy of quadriceps muscle cryosections from RRV-infected mice treated with IMP (RRV + IMP) or vehicle (RRV) at 9 dpi. Bars = 20 μm. (C) Quantification of RRV viral load in the quadriceps muscle of RRV-infected mice treated with IMP (RRV-IMP) or vehicle (RRV) at 5, 7, 9, and 15 dpi. Data (means ± SEM) are expressed as plaque-forming units (log_10_ PFU) per gram of tissue. Values that are not significantly different (ns) by Mann-Whitney U test (*P* < 0.05) are indicated. Data (means ± SEM; *n* = 5 mice per group) are from two independent experiments. N.D, not determined. (D) Tissue mRNA expression of matrix metalloproteinases *Mmp2*, *Mmp9*, and *Mmp12* in the quadriceps of RRV-IMP or RRV mice at 9 dpi and 15 dpi. Data (means ± SEM; *n* = 5 mice per group; three independent experiments) were normalized to *Gapdh* and shown as fold change relative to uninfected tissue. Values that are significantly different by one-way ANOVA and Dunnett’s posttest are indicated by asterisks as follows:*, *P* < 0.05; **, *P* < 0.01. Values that are not significantly different (n.s.) are indicated. (E) Total CCL2, IFN-γ, and TNF-α protein concentration in quadriceps homogenates from RRV-IMP, RRV, and uninfected (PBS) mice at 9 dpi and 15 dpi. Data (means ± SEM; *n* = 4 or 5 mice per group) are from two independent experiments. *, *P* < 0.05 by one-way ANOVA and Dunnett’s posttest. (F) Confocal microscopy of quadriceps muscle cryosections of RRV-IMP and RRV mice at 15 dpi. Sections were stained with Hoechst 33258 (nuclei), anti-desmin antibody, and phalloidin. The images shown are representative images (four mice per group and two independent experiments). Bars = 50 μm. (G) Quantification of desmin-positive phalloidin-positive myofibers in 30-μm-thick cryosections of quadriceps muscle from RRV-IMP or RRV mice at 15 dpi. 3D voxel thresholding analysis was used to quantify expression of desmin on myofibers. Data (means ± SEM; *n* = 3 mice per group) are from two independent experiments. ****, *P* < 0.0001 by Mann-Whitney U test.

Since our findings indicate that monocytes recruited at 8 dpi are more likely to convert to a CX_3_CR1^+^ phenotype and could promote tissue repair, we asked whether modulating the infiltration of inflammatory monocytes up until 8 dpi would favor the accumulation of this MP subset in the muscle as inflammation resolves. We used immune-modifying microparticles (IMP) that were shown to alter IM infiltration and limit IM-induced tissue damage in a model of WNV encephalitis. IMP are negatively charged, submicrometer microparticles with high avidity for the scavenger receptor MARCO (macrophage receptor with collagenous structure) expressed on the surfaces of inflammatory monocytes. Phagocytosis of IMP by IM is followed by their sequestration in the spleen, thereby significantly reducing the number of IM immigrating into the site of inflammation (23). IMP were administered intraperitoneally (i.p.) to WT RRV-infected mice daily from 4 dpi to 8 dpi, and the mice were monitored daily for disease. IMP treatment significantly reduced disease signs, with mice developing milder hind limb dysfunction by 9 dpi and recovering faster than their untreated counterparts (Fig. 4A). Confocal microscopy showed reduced Ly6C^+^ and CD11b^+^ cell infiltration in the quadriceps muscle of IMP-treated mice by 9 dpi (Fig. 4B). Interestingly, IMP-mediated modulation of IM infiltration had no effect on RRV replication in the muscle at the preacute (5 dpi), onset (7 dpi), acute (9 dpi), or recovery (15 dpi) phase of the disease (Fig. 4C). In addition, mRNA expression of matrix metalloproteinases (MMP) *Mmp2* was significantly upregulated at 9 and 15 dpi in IMP-treated mice compared to untreated RRV mice, and expression of *Mmp9* and *Mmp12* was significantly higher at 9 dpi in IMP-treated mice (Fig. 4D). Next, we asked whether IMP treatment affected local proinflammatory cytokine production. At 9 and 15 dpi, there was no difference in the levels of CCL2 or tumor necrosis factor alpha (TNF-α) protein in the muscle of IMP-treated mice compared to untreated mice, but gamma interferon (IFN-γ) was significantly reduced in the tissue of IMP-treated mice at 9 dpi, but not 15 dpi (Fig. 4E). To assess the difference in myofiber damage and regeneration between IMP-treated and untreated mice, muscle cryosections were immunolabeled for desmin, a marker specific for regenerating myofibers, and phalloidin, which labels all myofibers (Fig. 4F). Quantification of desmin-expressing myofibers revealed a significantly higher desmin expression in IMP-treated mice compared to untreated mice (Fig. 4G), and this difference was not seen in actin-labeled myofibers, indicating a higher proportion of regenerating myofibers in the muscle of IMP-treated mice.

### Modulation of CD11b^hi^ Ly6C^hi^ IM using IMP enhances the establishment of CX_3_CR1^+^ MP in the muscle.

**FIG 5:**
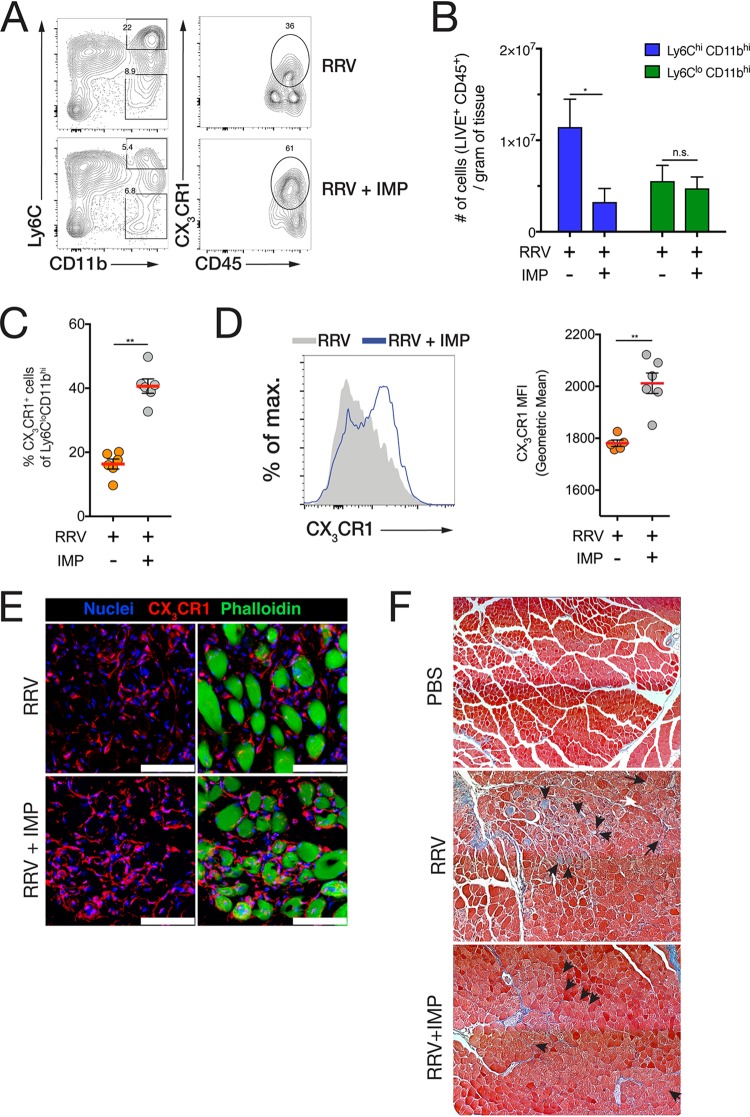
IMP treatment of RRV-infected mice promotes the establishment of CX_3_CR1^+^ macrophages associated with muscle tissue repair upon recovery. (A) Representative flow cytometry plots with gating of CD11b^hi^ Ly6C^hi^ inflammatory monocytes (IM) and CX_3_CR1^+^ CD11b^hi^ Ly6C^lo^ macrophages (MP) in infected quadriceps tissue of RRV-infected mice (RRV) and RRV-infected, IMP-treated mice (RRV-IMP) at 15 dpi. The parent population frequency is shown in the gates. Data (means ± SEM; *n* = 4 or 5 mice per group) are from three independent experiments. (B) Numbers of CD11b^hi^ Ly6C^hi^ and CD11b^hi^ Ly6C^lo^ cells in the quadriceps of RRV and RRV-IMP mice at 15 dpi. Data (means ± SEM; *n* = 4 or 5 mice per group) are from three independent experiments. *, *P* < 0.05 by Mann-Whitney U test; n.s., not significantly different by Mann-Whitney U test. (C) Percentage of CX_3_CR1^+^ cells within the CD11b^hi^ Ly6C^lo^ population in the quadriceps of RRV and RRV-IMP mice at 15 dpi. Data (means ± SEM; *n* = 5 mice per group) are from three independent experiments. **, *P* < 0.01 by Mann-Whitney U test. (D) Histogram overlay of CX_3_CR1 fluorescence intensity in CD11b^hi^ Ly6C^lo^ cells isolated from the quadriceps muscle of RRV and RRV-IMP mice. (Right) Geometric mean fluorescence intensity (MFI) of CX_3_CR1 expression in CD11b^hi^ Ly6C^lo^ cells in the quadriceps of RRV and RRV- IMP mice at 15 dpi. Data (means ± SEM; *n* = 5 mice per group) are from three independent experiments. **, *P* < 0.01 by Mann-Whitney U test. (E) Immunofluorescence staining of muscle tissue cross-sections (14-μm) RRV and RRV-IMP mice at 15 dpi. Sections were stained with Hoechst 33258 (nuclei), anti-CX_3_CR1 antibody, and phalloidin. Bars = 40 μm. (F) Masson trichrome immunohistochemical staining of paraffin-fixed sections of quadriceps muscle from uninfected (PBS), RRV-infected, and RRV-infected, IMP-treated C57BL/6 mice at 15 dpi. Black arrows denote fibrotic myofibers and collagen deposition. Data (means ± SEM; *n* = 4 mice per group) are from two independent experiments.

Having showed that CX_3_CR1^+^ MP established after 8 dpi were important for muscle tissue repair, we asked whether IMP treatment was associated with an increase in the number or proportion of CX_3_CR1^+^ MP in the muscle during resolution. At 15 dpi, the CD11b^hi^ Ly6C^hi^ IM infiltrate was reduced in the muscle of IMP-treated mice, but no difference in the number of CD11b^hi^ Ly6C^lo^ cells was observed (Fig. 5A and B). However, the proportion of CX_3_CR1^+^ cells within the CD11b^hi^ Ly6C^lo^ population was significantly higher in IMP-treated mice (Fig. 5C), and CD11b^hi^ Ly6C^lo^ MP displayed higher surface CX_3_CR1 expression in the muscle of IMP-treated mice (Fig. 5D). IMP treatment did not affect the number of CD4^+^, CD8^+^, and γδ T cell receptor-positive (γδTCR^+^) T cells, NK cells, neutrophils, and dendritic cells in the muscle tissue of RRV-infected mice at 9 dpi (see Fig. S1 in the supplemental material) and had no effect on T cell and NK cell activation (Fig. S2). In addition, confocal microscopy confirmed that CX_3_CR1^+^ macrophages were more abundant around the muscle fibers of IMP-treated mice at 15 dpi (Fig. 5E), and Masson trichrome histochemical staining showed more severe tissue damage and collagen deposition at 15 dpi in untreated, RRV-infected mice compared to IMP-treated mice (Fig. 5F).

10.1128/mBio.03353-19.1 FIG S1 Quantification of cellular infiltrates in the quadriceps muscle of IMP-treated and RRV-infected mice at 9 dpi. Tissues were harvested and homogenized, and CD4^+^ and CD8^+^ T cells (LIVE CD45^+^ CD3^+^), NK cells (LIVE CD45^+^ CD3^−^ NK1.1^+^), γδTCR^+^ T cells (LIVE CD45^+^ CD3^+^ γδTCR^+^), dendritic cells (LIVE CD45^+^ CD11b^lo^ CD64^−^ CD24^hi^ CD11c^+^ migratory dendritic cells [DCs] and LIVE CD45^+^ CD11b^hi^ Ly6C^hi^ CX_3_CR1^+^ CD64^+^ monocyte-derived DCs) and neutrophils (LIVE CD45^+^ CD11b^+^ Ly6C^int^ Ly6G^+^) were enumerated as described in Materials and Methods. Statistical significance was defined by a *P* value of 0.05 and assessed via multiple *t*-test analysis with a Holm-Sidak correction for multiple comparisons. n.s., not significant. Download FIG S1, PDF file, 0.1 MB. Copyright © 2020 Zaid et al. 2020 Zaid et al. This content is distributed under the terms of the Creative Commons Attribution 4.0 International license.

10.1128/mBio.03353-19.2 FIG S2 Quantification of activated T cells and NK cells in the quadriceps muscle of IMP-treated and RRV-infected mice at 9 dpi. Tissues were harvested and homogenized, and CD4^+^ and CD8^+^ T cells expressing CD69 or CCR6, and NK cells expressing CD69 were enumerated as described in Materials and Methods. Statistical significance was defined by a *P* value of 0.05 and assessed via multiple *t*-test analysis with a Holm-Sidak correction for multiple comparisons. n.s., not significant. Download FIG S2, PDF file, 0.7 MB. Copyright © 2020 Zaid et al. 2020 Zaid et al. This content is distributed under the terms of the Creative Commons Attribution 4.0 International license.

### Modulation of IM infiltration alters the transcriptional profile of infiltrating monocytes and tissue MPs upon resolution.

**FIG 6:**
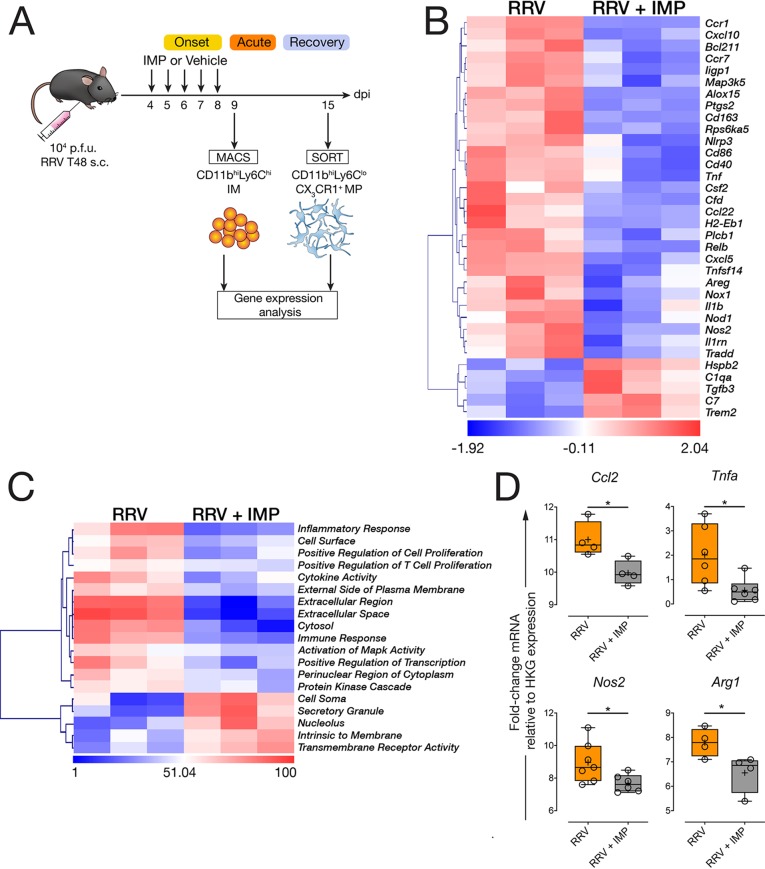
IMP treatment alters the transcriptional profile of CD11b^hi^ Ly6C^hi^ IM and CX_3_CR1^+^ CD11b^hi^ Ly6C^lo^ MP. (A) Schematic describing the isolation of CD11b^hi^ Ly6C^hi^ IM at 9 dpi and CX_3_CR1^+^ CD11b^hi^ Ly6C^lo^ MP at 15 dpi from the quadriceps muscle of RRV-infected, and RRV-infected, IMP-treated mice. Cells were isolated by magnetically activated cell sorting (MACS) at 9 dpi or by fluorescence-activated cell sorting (SORT) at 15 dpi for Nanostring analysis using the Mouse Inflammation gene set or by qRT-PCR. Cells isolated from quadriceps (six mice per group) were pooled into three duplicates; data are representative of two independent experiments. (B) Heatmap showing differentially expressed genes in CD11b^hi^ Ly6C^hi^ IM isolated from the muscle of RRV and RRV-IMP mice at 9 dpi. Data are target gene expression relative to housekeeping gene expression panel, and heat map coloring is based on row *z*-score. Data points represent two pooled C57BL/6 WT mice. Hierarchical clustering is unsupervised. Counts were normalized and log_2_ transformed, *P* values above *P* = 0.05 (two-tailed *t* test) were excluded from the analysis. (C) Heatmap showing pathway activation using unsupervised hierarchical clustering in CD11b^hi^ Ly6C^hi^ IM isolated from the muscle of RRV and RRV-IMP mice at 9 dpi. Heatmaps were generated following normalization of digital RNA counts and *z*-transformed. *P* values above *P* = 0.05 (two-tailed *t* test) were excluded from the analysis. (D) Quantitative real-time PCR analysis of *Ccl2*, *Tnfa*, *Nos2*, and *Arg1* gene expression in CX_3_CR1^+^ CD11b^hi^ Ly6C^lo^ MP isolated from the muscle of RRV and RRV-IMP mice at 15 dpi. Data are expressed as fold change mRNA expression relative to normalized housekeeping gene expression. Data (four to six mice; data for two mice pooled for each sample) are representative of two pooled independent experiments. *, *P* < 0.05 by Mann-Whitney U test.

Next, we asked whether IMP treatment functionally altered IM during the acute phase and whether this change subsequently altered CX_3_CR1^+^ MP at a transcriptional level during resolution. We isolated CD11b^hi^ Ly6C^hi^ IM from the muscle tissue of RRV-infected mice treated with IMP (or vehicle) at 9 dpi and performed differential gene expression (DGE) and pathway activation analysis using a Nanostring array (Fig. 6A). Analysis revealed that 34 genes were differentially expressed (*P* < 0.05) in IM isolated from the muscle of IMP-treated mice compared to those isolated from untreated mice at 9 dpi (Fig. 6B). Modulation of IM using IMP was associated with the downregulation of 29 genes during the acute phase, including *Cxcl10*, *Tnf*, *Cxcl5*, *Nos2*, *Il1b*, and *Nlrp3*, whereas 5 genes were found to be upregulated in muscle IM isolated from IMP-treated mice, including *Trem2*, *Tgfb3*, and *C1qa* (Fig. 6B). Pathway activation analysis revealed that inflammatory response, immune response, and cytokine activity pathways were strongly activated in IM isolated from the muscle of untreated, RRV-infected mice compared to those isolated from IMP-treated mice, indicating a shift away from the proinflammatory profile found in acute RRV-induced muscle inflammation (Fig. 6C). Next, we asked whether IMP treatment of RRV-infected mice subsequently led to a skewed transcriptional profile of CX_3_CR1^+^ macrophages in the resolution phase at 15 dpi. We sorted CX_3_CR1^+^ CD11b^hi^ Ly6C^lo^ MP from the muscle RRV-infected mice and found that IMP treatment was associated with a significant downregulation of *Ccl2*, *Tnfa*, and *Nos2* gene expression compared to CX_3_CR1^+^ MP isolated from the muscle of untreated mice (Fig. 6D). Interestingly, the expression of *Arg1*, a gene typically associated with M2-polarized macrophages, was also significantly downregulated in CX_3_CR1^+^ MP isolated from the muscle of IMP-treated mice at 15 dpi (Fig. 6D).

## DISCUSSION

Arthritis and myositis caused by arboviruses are debilitating for a large portion of the affected populations (1, 31), and in the absence of an effective vaccine, the only available approaches are palliative solutions using nonsteroidal anti-inflammatory drugs. Here, using a mouse model of RRV-induced myositis, we showed that muscle tissue inflammation follows a bimodal inflammatory process driven by CD11b^hi^ Ly6C^hi^ inflammatory monocytes (IM), culminating in a recovery phase associated with the establishment of a CX_3_CR1^+^ CD11b^hi^ Ly6C^lo^ macrophage (MP) population. Development of the latter, concomitant with muscle tissue repair, was consistent with an M2-like, repair-promoting macrophage phenotype.

It was previously shown that alternatively activated, or M2-like MP could promote muscle tissue regeneration in sterile injury models (32, 33), and CX_3_CR1 deficiency was associated with impaired muscle tissue repair (34), in line with our observations that recovery of muscle tissue integrity was delayed in CX_3_CR1-deficient mice infected with RRV—though it must be noted that in the context of sterile muscle injury, regeneration was not solely dependent on CX_3_CR1^+^ MP (35). During viral infection, tissue damage and subsequent repair is accompanied by the differential temporal deployment of a wide range of soluble factors required to eradicate and resolve infection as well as the influx of various cellular elements of both adaptive and innate responses, complicating this process considerably. It was previously shown that monocytes/macrophages were the main drivers of inflammation in RRV disease (RRVD), and clodronate-liposome depletion helped alleviate tissue infiltration (10). However, this study did not explore the role played by MP in muscle tissue repair, and it has since been shown that clodronate-mediated monocyte/macrophage lineage depletion is associated with impaired muscle tissue regeneration (36), indicating that broad depletion of this lineage would likely result in the removal of both inflammatory and reparative populations. Thus, in a viral setting, this may reduce inflammation but may impede virus clearance and in turn delay tissue restoration/repair. This is corroborated by a study showing that CCR2^+^ monocytes were essential in controlling RRV infection in a type I IFN-dependent manner, providing evidence that IM are required in the early stages to limit excessive viral replication, despite their role in inducing tissue damage and, incidentally, trafficking virus to distant anatomical sites (11). The role of M2-like MP in tissue repair and regeneration has been the focus of several studies (19, 31, 37, 38); however, few studies have focused on inflammation resulting from viral infections. Characterization of M2-like MP has identified key markers such as *Chi3l3/Ym1*, *Relma*, *Il10*, and *Tgfb*, although more comprehensive panels of specific markers have been established recently (39, 40). Our data indicate that the timing of monocyte recruitment to the site of inflammation in RRV-induced myositis is critical for the subsequent accumulation of M2-like CX_3_CR1^+^ MP during recovery. Monocyte recruitment to the muscle during the acute phase of inflammation (8 dpi) resulted in the accumulation of CD163^+^ CCR2^−^ macrophages, in contrast to those recruited during the preacute phase (4 dpi). This suggested that targeting IM infiltration during onset and peak disease could be beneficial should it promote the accumulation of tissue repair-associated MP in the muscle.

In some disease settings, the initial IM inflammation is pathogenic and can be modulated using immune-modifying microparticles, or IMP, to ameliorate disease outcome. IMP have been shown to limit IM-driven brain inflammation caused by WNV, a mosquito-borne flavivirus, and this effect was also observed in experimental models of inflammatory bowel disease and ischemia-reperfusion injury (41) and more recently in a model of herpes simplex virus keratitis (42). RRV-infected mice treated with IMP in the early phase of muscle inflammation (4 dpi to 8 dpi) had significantly reduced clinical scores (e.g., hind limb dysfunction, foot dragging) and muscle tissue damage. IMP treatment reduced the number of IM infiltrating into the muscle tissues in the acute phase without affecting the number or activation state of other immune cells found in the tissue. Of note, administration of IMP from 7 dpi alone was less effective in ameliorating disease compared to the 4 dpi to 8 dpi, 5-day regimen (see Fig. S3 in the supplemental material), reinforcing the notion that the timing of modulation is critical. Importantly, IMP treatment did not increase the viral load in the muscle tissue, which may be because IMP treatment did not prevent all IM from immigrating into the virus-infected tissue. The number of IM recorded at the inflammatory focus in IMP-treated mice varies between 25% (Fig. 5B) and 50% (23) of those seen in untreated mice, suggesting that IMP treatment is tunable, but that antiviral IM that migrate to the muscle in the early phase during IMP treatment may be sufficient to control the viral load. Therefore, the optimal timing of IMP treatment may be confined to the preacute phase of inflammation (e.g., up until 8 dpi), after which time monocytes recruited to the muscle are transcriptionally programmed to convert to CX_3_CR1^+^ MP.

10.1128/mBio.03353-19.3 FIG S3 RRV disease score for mice infected with RRV with two IMP treatment regimens. C57BL/6 mice were infected with RRV, and IMP were administered i.p. as described in Materials and Methods. Mice were treated with IMP for 5 days (orange symbols) between 4 dpi and 8 dpi or for 1 day (magenta symbols) at 8 dpi. Mice were monitored for disease progression according to clinical signs described in Materials and Methods. Data represent the means (± SEM; *n* = 5 mice per group) and are representative of two independent experiments. ***, *P* < 0.005 by two-way ANOVA (IMP 1x versus IMP 5x), with Geisser Greenhouse correction and a Dunnett’s multiple-comparison posttest. Download FIG S3, PDF file, 0.6 MB. Copyright © 2020 Zaid et al. 2020 Zaid et al. This content is distributed under the terms of the Creative Commons Attribution 4.0 International license.

In this study, IMP treatment was associated with increased formation of CX_3_CR1^+^ MP in the muscle, and this was accompanied by improved tissue regeneration. In contrast, the effect of IMP modulation was milder on the local production of proinflammatory CCL2 and TNF-α, suggesting that infiltrating cells other than those targeted by IMP may be contributing to the proinflammatory environment. IMP treatment lead to an upregulation of matrix metalloproteinase (MMP) *Mmp2*, *Mmp9* and *Mmp12*; MMPs are essential for tissue remodeling and are activated at different stages following tissue injury (43–45), suggesting that the reduction in IM infiltration as a result of IMP modulation in turn helps local tissue microenvironment regulate transcriptional activity to promote tissue repair.

CX_3_CR1 expression regulates all stages of macrophage development and is a classical marker for patrolling monocytes, intestinal macrophages, and microglia (46, 47). As a key marker of microglia, CX_3_CR1 is critical in the regulation of inflammatory processes in the brain (48, 49), and it is generally understood that M2-like, tissue repair-associated macrophages express CX_3_CR1 (17, 37) and depend on IL-4 and IL-10, both key effectors of M1-to-M2 monocyte transition (50, 51). We show that over the course of RRV disease, the tissue microenvironment shifts from an acute, proinflammatory milieu mediated by high *Ccl2*, *Cxcl10*, *Nos2*, and *Tnfa* expression toward a tissue repair environment during resolution where *Il10*, *Tgfb*, and *Mmp12* expression is prominent, suggesting that muscle CX_3_CR1^+^ MP are formed in a local M2-like tissue microenvironment during resolution. M2-like macrophages have previously been characterized by their expression of arginase gene *Arg1* (39, 40), but in RRV-induced myositis, two studies showed that specific depletion of Arg-1-expressing myeloid cells enhanced viral clearance and reduced pathology (52, 53), and our findings showing that *Arg1* mRNA is highly expressed in the muscle during the acute phase of the disease (6 to 9 dpi) are consistent with these findings. Importantly, our data also show *Arg1* mRNA expression is downregulated in CX_3_CR1^+^ MP isolated from the muscle of IMP-treated mice at 15 dpi, thus highlighting the possibility that IMP may promote the local expansion of tissue repair-associated macrophages by indirectly dampening *Arg1* expression in these cells.

Transcriptional analysis of infiltrating IM in the acute phase (9 dpi) revealed that IMP helped dampen proinflammatory *Il1b*, *Nos2*, *Tnfa*, *Nlrp3*, and *Csf2* expression, which have all been shown to be important host factors in alphavirus-induced disease, thus contributing to a shift against proinflammatory pathways associated with acute inflammation (54–58). Of note, IFN-γ-induced *Cxcl10* gene expression was downregulated in IM isolated from the muscle of IMP-treated mice, and together with the reduced IFN-γ protein levels we observed in the muscle, our data point toward an important role for IFN-γ and CXC chemokine ligand 10 (CXCL10)-CXC chemokine receptor 3 (CXCR3) pathways in RRV-induced myositis.

Gene expression of CX_3_CR1^+^ MP isolated from the muscle of IMP-treated mice in the recovery phase (15 dpi) was consistent with an M2-like phenotype, with reduced *Tnfa*, *Ccl2*, and *Nos2* expression compared to CX_3_CR1^+^ MP isolated from untreated mice. Taken together, these data strongly suggest that modulation of IM during the acute phase of the disease not only reduces IM tissue infiltration but also shapes the transcriptional program of CX_3_CR1^+^ MP toward an M2-like, tissue repair-promoting phenotype. This is important because M2-polarized macrophages have been shown to promote skeletal muscle regeneration by stimulating muscle progenitor cells to commit to myocyte differentiation (32, 33) and our work indicates that M2-like CX_3_CR1^+^ MP may likewise be implicated in this process, though further studies are needed to establish a link between MP and myocyte differentiation in RRV myositis.

In arthritogenic alphavirus-induced myositis, muscle MPs may commit to a distinct M2-like transcriptional program in the wake of acute, IM-driven tissue inflammation, allowing their establishment and maintenance after viral clearance and resolution of inflammation. Our data also show that timely modulation of IM infiltration not only abates monocyte infiltration and tissue damage but may enable a transcriptional program that facilitates the accumulation of M2-like CX_3_CR1^+^ MP during resolution. In conclusion, as systemic depletion of inflammatory monocytes is neither desirable—given their importance in viral clearance—nor practical from a clinical perspective, our work complements current knowledge by showing that proinflammatory properties of specific immune cell subsets can be modulated using particles such as IMP to reduce inflammation and enhance tissue repair.

## MATERIALS AND METHODS

### Mice and infections.

Animal experiments were approved by the Animal Ethics Committee of Griffith University (AEC GLY06/13, GLY19/17, and GLY06/18). All procedures involving animals conformed to the *Australian Code of Practice for the Care and Use of Animals for Scientific Purposes* (59). Wild-type C57BL/6 mice were obtained from the Animal Resource Centre (Perth, Australia), *csf1r^eGFP^* reporter mice were purchased from the University of Queensland, and CX_3_CR1^+/GFP^ mice were obtained from the University of Melbourne. Mice were housed and bred in the Bioscience Resource Facilities of Griffith University (Gold Coast, Australia). For the Ross River virus (RRV) mouse model, 21 day-old C57BL/6 male and female mice, of equal distribution, were inoculated subcutaneously (s.c.) below the right forelimb with 10^4^ PFU of RRV in 50 μl, and mock-infected mice were inoculated with 50 μl of phosphate-buffered saline (PBS) diluent alone. Mice were scored according to a cumulative and progressive clinical disease matrix described previously as follows: 0 for no disease, 1 for ruffled fur, 2 for lethargy and weak hind limb grip, 3 for mild hind limb dysfunction (delicate walking on hind limbs and/or near loss of gripping ability), 4 for moderate hind limb dysfunction (labored walking pattern with splayed hind legs and loss of gripping ability), and 5 for complete loss of hind limb function (complete hind limb dragging and exclusive forelimb traction). Negatively charged, polylactic co-glycolic acid immune-modifying microparticles (IMP) (500-nm diameter) (Phosphorex, Hopkinton, MA, USA) were administered intraperitoneally (i.p.) (15 mg/kg of body weight) to C57BL/6 mice once daily from day 4 postinfection until day 8 postinfection, in a volume of 200 μl of sterile PBS.

### Virus.

The T48 prototype strain of Ross River virus (isolated in Townsville, Queensland, Australia, in 1959 from Aedes vigilax mosquitoes [60]) was generated via *in vitro* transcription of a SacI-linearized pRR64 plasmid (61) (established and provided by Richard Kuhn, Purdue University, USA) encoding the full-length T48 clone. After 10 consecutive passages in the brains of suckling mice (62), the virus was passaged in Vero cells (ATCC CCL-81) twice to generate stocks that were later propagated in Vero cells for use in the RRV pathogenesis model as described previously (24). The T48 strain is specifically mouse virulent, unlike other strains isolated elsewhere in Australia (e.g., RRV NB5092, from Nelson Bay, New South Wales) which displays no virulence in mice (63). Virus was titrated by plaque assay on Vero cells and diluted to the required concentration (10^4^ PFU) in sterile, endotoxin-free PBS for animal experiments.

### Flow cytometry.

Mice were euthanized at designated time points following RRV infection, and quadriceps tissue was harvested following transcardial perfusion with ice-cold PBS. Quadriceps muscles were weighed, minced, and digested in type IV collagenase (1 mg/ml in RPMI 1640 medium plus 2% fetal calf serum [RPMI + 2% FCS]; Worthington Biochemical Co., NJ, USA) supplemented with 1 mg/ml DNase type I (Sigma-Aldrich USA, Inc.) at 37°C for 1 h. Digested muscle was resuspended in RPMI + 10% FCS and successively filtered through 70-μm and 30-μm nylon meshes. Cells were washed in staining buffer (PBS with 2% FCS and 5 mM EDTA) and labeled with fluorochrome-conjugated antibodies from eBioscience (San Diego, CA, USA), BD Biosciences (San Jose, CA, USA), Biolegend (San Diego, CA, USA), or Santa Cruz Biotechnology (Dallas, TX, USA). Cells were blocked with CD16/32 antibody (2.4G2) and labeled with antibodies against mouse CD11b (M1/70), CD4 (RM.4-5), CD3 (17A2), CD8 (53.6-7), CD69 (H1.2F3), CD64 (X54-5/7.1), CCR6 (140706), NK1.1 (PK136), CD11c (N418), CD24 (M1/69), Ly6C (HK1.4), CD45 (30-F11), CX_3_CR1 (SAO11F11), γδ T cell receptor (γδTCR) (GL-3), CCR2 (SA203G11), Ly6G (1A8), and CD163 (polyclonal goat IgG; Santa Cruz). Dead cells were excluded using LIVE/DEAD Near Infrared (Thermo Fisher, Australia) cell viability dye. Counting beads (Sphero calibration beads [catalog no. 556296; BD Biosciences]) were counted using a hemocytometer and added to each sample prior to acquisition. Cells were examined with a BD LSR II Fortessa instrument, and data were analyzed using FlowJo software (version 10.2; TreeStar, Inc.) and plotted using GraphPad Prism v8 (GraphPad Software, LLC).

### Cell sorting and total RNA extraction.

CX_3_CR1^+^ CD11b^hi^ Ly6C^lo^ cells were sorted from homogenized quadriceps tissue from IMP-treated or vehicle-treated RRV-infected mice at 15 days postinfection (dpi). Muscle tissue was processed as described above and prepared for sorting in sorting buffer (RPMI + 10% FCS + 5 mM EDTA). Cells were sorted in a BD Aria III FACSSorter to a purity of 95 to 98% and collected in RNase-free Eppendorf tubes (gating strategy shown in Fig. S4 in the supplemental material). Alternately, CD11b^hi^ Ly6C^hi^ inflammatory monocytes were isolated from the muscle tissue of IMP-treated or vehicle-treated RRV-infected mice at 9 dpi by negative magnetic separation using a custom-designed magnetically activated cell sorting (MACS) Miltenyi monocyte isolation kit (catalog no. 130-100-629) supplemented with biotinylated anti-CD31 antibody. Single-cell suspensions were obtained from collagenase-digested muscle tissue, labeled with bead-conjugated antibodies, and passed through a Miltenyi MACS LS column. Total RNA was isolated from sorted cell pellets using iScript sample preparation buffer (Bio-Rad, Gladesville, New South Wales [NSW], Australia) according to the manufacturer’s instructions and processed for Nanostring nCounter analysis or quantitative real-time PCR.

10.1128/mBio.03353-19.4 FIG S4 Gating strategy employed for flow cytometry analysis and cell sorting of mononuclear cells and Ly6C^lo^ CX_3_CR1^+^ macrophages. Cells were isolated from the muscle tissue as indicated in the manuscript and labeled with fluorochrome-conjugated antibodies. Following dead cell exclusion, Ly6G^+^ neutrophils were gated out prior to setting a CD45^+^ leukocyte gate and subsequent Ly6C and CD11b gates. Flow cytometry plots are representative of at least four independent flow cytometry experiments and three cell sorting experiments. Download FIG S4, PDF file, 0.6 MB. Copyright © 2020 Zaid et al. 2020 Zaid et al. This content is distributed under the terms of the Creative Commons Attribution 4.0 International license.

### Confocal microscopy.

Quadriceps muscles were collected, fixed in 4% paraformaldehyde (PFA), and dehydrated in 30% (wt/vol) sucrose (in PBS). Cryosections that were 14 μm thick were permeabilized in acetone, blocked with 5% bovine serum albumin (BSA) for 1 h and immunolabeled with antibodies against desmin (ab32362; Abcam, Cambridge, UK), CX_3_CR1 (ab31331; Abcam, Cambridge, UK), CD68 (MCA1957GA; Bio-Rad, Gladesville, NSW, Australia), CD11b (M1/70; BD Biosciences, San Jose, CA, USA), or Ly6C (HK1.4; Biolegend, San Diego, CA, USA), and detected using Alexa Fluor 488-conjugated anti-rat and Alexa Fluor 568-conjugated anti-rabbit (Thermo Fisher, Australia). Sections were counterstained with Alexa Fluor 647-conjugated phalloidin (Thermo Fisher, Australia), Hoechst 33258 (Sigma-Aldrich USA, Inc.), and mounted with Prolong Gold Antifade (Thermo Fisher, Australia). Images were acquired on an Olympus FV1000 and FV3000 confocal microscope and processed using Imaris 9.2 (Bitplane). Three-dimensional (3D) thresholding analysis of desmin-positive myofibers was performed using Imaris Surfaces function. Desmin-positive and phalloidin-positive myofibers were rendered as surface objects, thresholds were applied using the automated threshold function, and voxel area data were plotted for analysis. Data were generated from ten 30-μm-thick cryosections per group (four mice per group).

### Quantitative real-time PCR (qRT-PCR).

Total RNA extraction was performed using TRIzol reagent (Life Technologies, Australia) following the manufacturer’s instructions. Total RNA was quantified by using a NanoDrop 1000 spectrophotometer (Thermo Scientific, Victoria, Australia). Total RNA (10 ng/μl) was reverse transcribed using oligo(dT_15_) primers and Moloney murine leukemia virus (M-MLV) reverse transcriptase (Sigma-Aldrich USA, Inc.) and amplified using SYBR green Supermix (Bio-Rad, Gladesville, NSW, Australia) in a 12.5-μl reaction mixture volume with 10 ng cDNA. Real-time PCR was performed using Bio-Rad CFX96 touch real-time PCR detection system. Forward and reverse primer sequences purchased from Sigma-Aldrich are listed in Table S1 in the supplemental material. Primers for *Hprt*, *Gapdh*, *Arg1*, *Ifnb*, *Il1b*, *Tnf*, *Il6*, *Ccl2*, *Ccl3*, *Cxcl10*, and *Il10* genes were Quantitect Primer Assay probes (Qiagen, Australia). Fold change in gene expression was determined through ΔΔ*C_T_* (*C_T_* stands for threshold cycle), where ΔΔ*C_T_* = Δ*C_T_*^(infected)^ – Δ*C_T_*^(mock)^, while Δ*C_T_* = *C_T_*^(gene of interest)^ – *C_T_*^(housekeeping gene – HPRT)^ (HPRT stands for hypoxanthine phosphoribosyltransferase). The fold change for each gene was calculated as 2^-ΔΔCt^. Unsupervised hierarchical clustering, *z*-score transform, row clustering, and heatmap visualization was performed using Morpheus (https://software.broadinstitute.org/morpheus).

10.1128/mBio.03353-19.5 TABLE S1 List of murine gene primers (forward and reverse sequences) used for real-time quantitative polymerase chain reaction (qRT-PCR) as described in Materials and Methods. Download Table S1, PDF file, 0.05 MB. Copyright © 2020 Zaid et al. 2020 Zaid et al. This content is distributed under the terms of the Creative Commons Attribution 4.0 International license.

### nCounter gene expression analysis and statistics.

Sorted cells were counted and lysed in 5 μl iScript Sample Preparation buffer (catalog no. 170-8898; Bio-Rad, Gladesville, NSW, Australia) per the manufacturer’s instructions to yield a final concentration of 10,000 cells per microliter of lysis buffer. RNA target molecules were quantified using a nCounter mouse inflammation v2 gene panel (catalog no. LBL-10402-01; Nanostring Technologies, CA, USA), and samples were processed according to the nCounter gene expression protocol. Briefly, 5 μl of cell lysate, 8 μl of mastermix, and 2 μl of capture probe were used for hybridization. After 24-h hybridization at 65°C, excess probes were washed using a two-step magnetic bead-based purification system on an nCounter Prep-station instrument and immobilized in a sample cartridge for data collection. Data collection was performed on the nCounter digital analyzer. Background correction was performed via subtraction of negative-control probes, and normalization was done using a combination of positive-control normalization for technical variability and CodeSet Content normalization, which uses housekeeping genes, for assay input variability. Gene expression data were analyzed using the Advanced Analysis Module in the nSolver Analysis software (v. 4.0) from Nanostring Technologies (CA, USA) and TIGR Multi Experiment Viewer (http://mev.tm4.org). Raw data were normalized by subtracting the geometric mean plus 1 standard deviation of eight negative controls, while technical variation was normalized through internal positive controls. Data were corrected for input material via internal housekeeping genes. A transcript was considered not detected if its mean count was below the mean plus 1 standard deviation of the negative-control counts in more than 60% of samples. Counts above minimal negative threshold were *z*-transformed, and relative gene expression between IMP-treated and untreated groups was compared using a two-tailed *t* test. Genes with a differential expression at *P* < 0.05 were clustered for visual analysis using unsupervised hierarchical clustering and submitted to a false-discovery rate (FDR) analysis. Using pathway scores calculated in nSolver, we performed differential expression analysis and hierarchical clustering to compare 19 canonical immune pathways between treated and untreated groups. Statistical significance was set at *P* = 0.05, and a global significance score was determined by the square root of the mean signed squared *t* statistic for the genes within a gene set.

### Statistical analysis.

All data are shown as means ± standard errors of the means (SEM), where statistical analysis is required. Significance between the values for the experimental groups was determined by the *P* value (*P* < 0.05, *P* < 0.01, *P* < 0.001, or *P* < 0.0001, as indicated in the figure legends), using a Mann-Whitney U test or specific one-way analysis of variance (ANOVA) test with appropriate posttests as specified in the figure legends. No animals were excluded from the analysis, and animals were allocated to their respective groups randomly. Sample size was determined by power analysis. Male and female mice were used in equal distributions in each group, and mice were allocated to their respective groups prior to being weighed to avoid a body mass-dependent bias when allocating groups. Scoring of disease signs following viral infection was performed by two researchers, one researcher scored disease in a blind manner, one researcher scored disease in a nonblind manner. For comparison between vehicle- and IMP-treated groups, the disease score was analyzed by two-way ANOVA. Results of qRT-PCR of mouse specimens were analyzed by one-way ANOVA with Dunnett’s posttest. Longitudinal qRT-PCR analyses of mouse specimens were performed using one-way ANOVA with Dunnett’s posttest. All data were assessed for Gaussian distribution using the D’Agostino-Pearson normality test before analysis with these parametric tests. Statistical analyses were performed with GraphPad Prism (v8).
