# Supplementary figures and images for: Modulation of Monocyte-Driven Myositis in Alphavirus Infection Reveals a Role for CX3CR1+ Macrophages in Tissue Repair
Source: mBio. 2020 Mar 3;11(2):e03353-19. doi: 10.1128/mBio.03353-19 (PMC7064784; doi:10.1128/mBio.03353-19)

# Supplementary Fig. 1

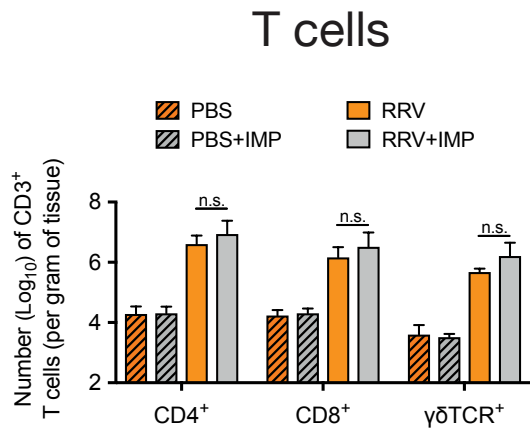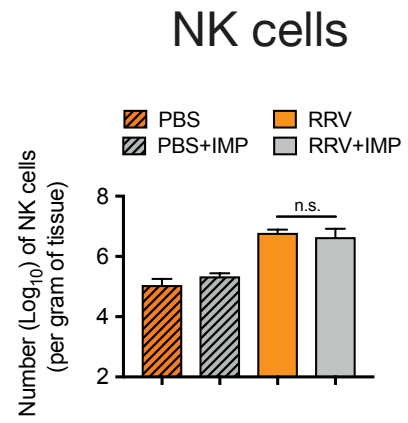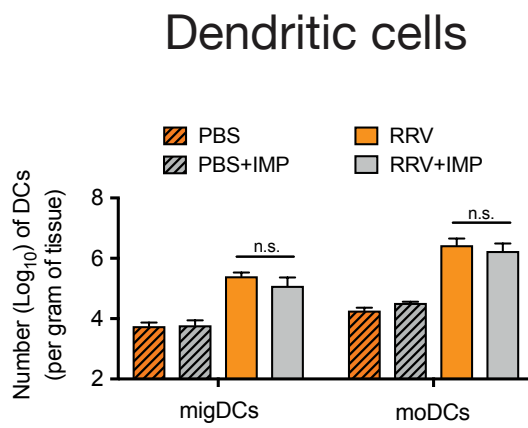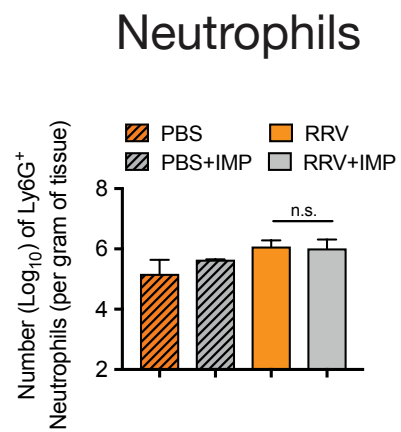

Supplement: FIG S1 [file mBio.03353-19-sf001.pdf]

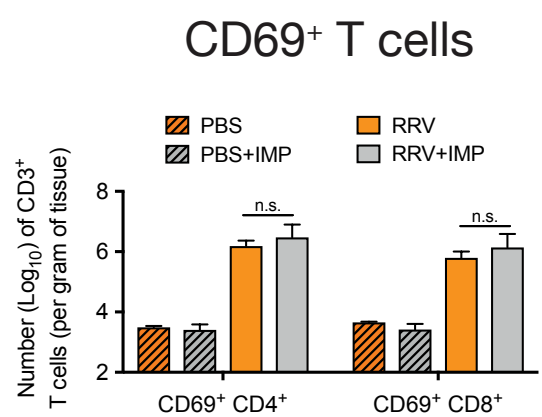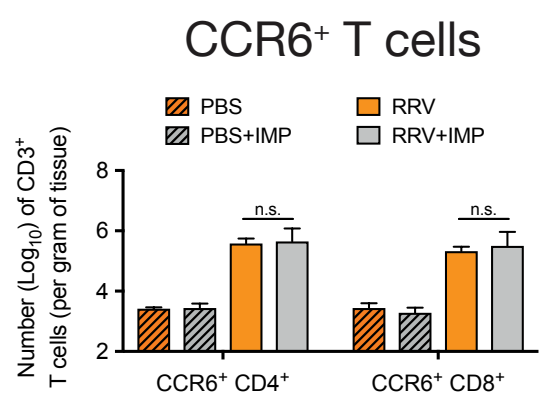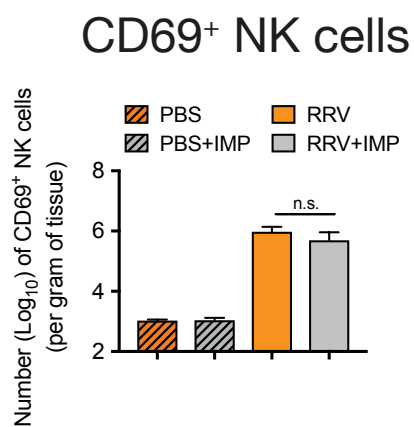

Supplement: FIG S2 [file mBio.03353-19-sf002.pdf]

# Supplementary Fig. 3

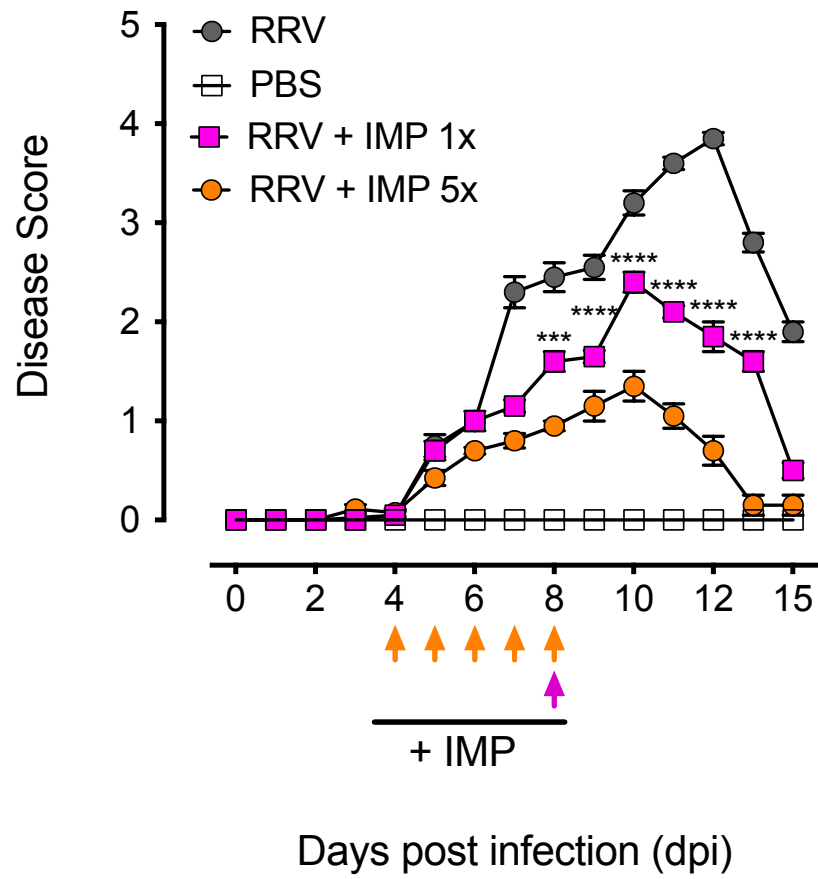

Supplement: FIG S3 [file mBio.03353-19-sf003.pdf]

# Supplementary Fig. 4

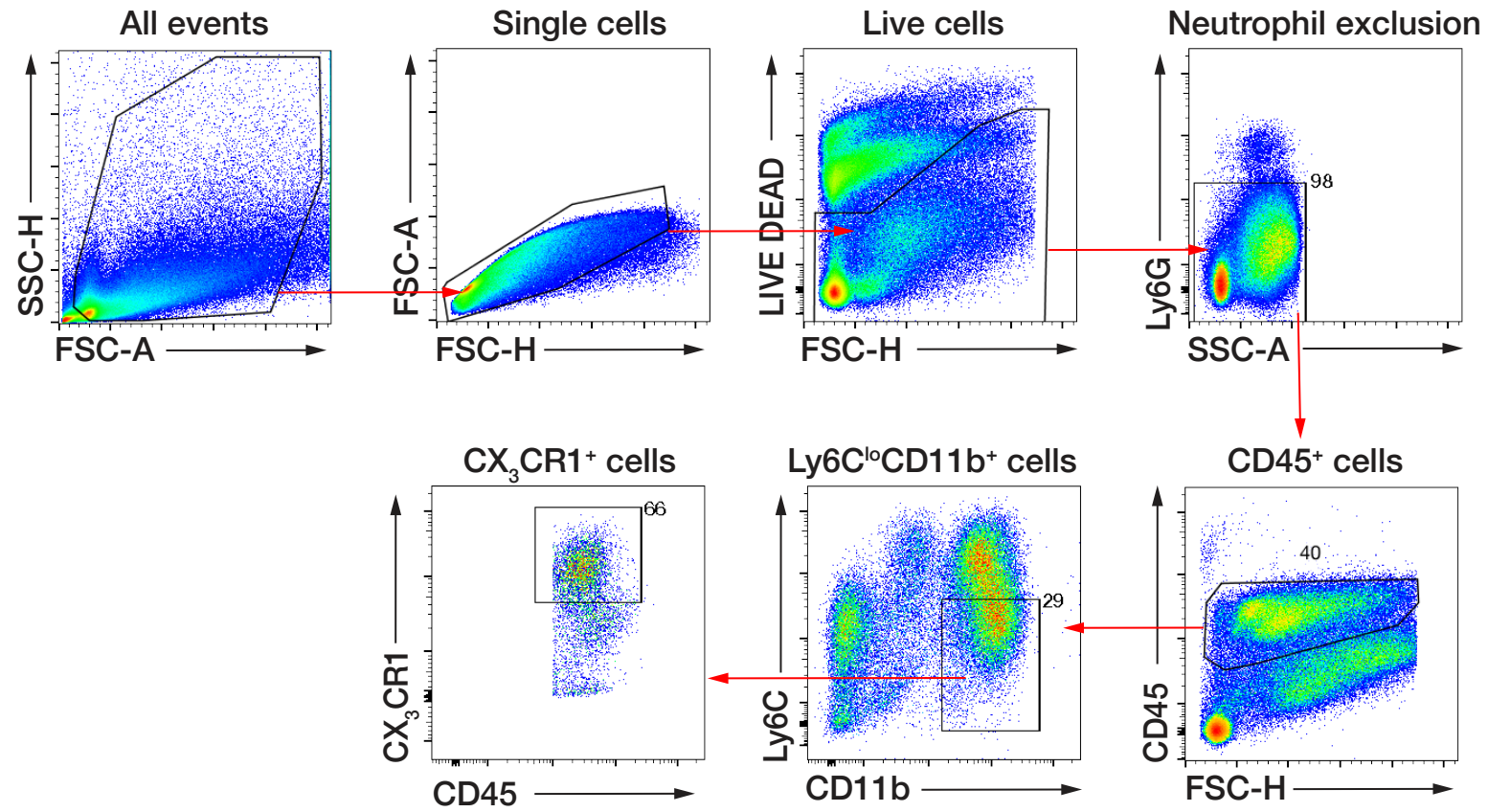

Supplement: FIG S4 [file mBio.03353-19-sf004.pdf]
