# Supplementary material for: Modulation of Monocyte-Driven Myositis in Alphavirus Infection Reveals a Role for CX3CR1+ Macrophages in Tissue Repair
Source: mBio. 2020 Mar 3;11(2):e03353-19. doi: 10.1128/mBio.03353-19 (PMC7064784; doi:10.1128/mBio.03353-19)
Supplement: TABLE S1 [file mBio.03353-19-st001.pdf]

Supplementary Table 1

Target gene	Forward	Reverse
Mrf4	GTGGCCAAGTGTTTCGGATC	AAAGGCGCTGAAGACTGCTG
Myf5	ACAGCAGCTTTGACAGCATC	AAGCAATCCAAGCTGGACAC
Myog	TGAGCATTGTCCAGGCCAG	GCTTCTCCCTCAGTGTGGCT
Myod1	CGCTCCAACTGCTCTGATG	TAGTAGGCGGTGTCGTAGCC
Nos2	ACATCGACCCGTCCACAGTAT	CAGAGGGGTAGGCTTGTCTC
Chi3l3	TCACAGGTCTGGCAATTCTTCTG	TTTGTCCTTAGGAGGGCTTCCTC
Timp-1	CCTTGCAAACTGGAGAGTGACA	AGGCAAAGTGATCGCTCTGGT
Mmp12	TTAACCCCAGCACATTTCGC	ACTGAATGTTACGTATGTCATCAGCA
Mmp9	CCCAAAGACCTGAAAACCTCC	TTCTCTCCCATCATCTGGGC
Mmp2	AGGAGCTCTATGGGCCCTCC	TCCTGTTTGCAGATCTCCGG
Tgfb1	CAACGCCATCTATGAGAAAACC	AAGCCCTGTATTCCGTCTCC
Pax3	CCAACCATATCCGCCACAA	TCTTAGAGACGCAACCATGGG
Ccl7	AATGCATCCACATGCTGCTA	CTTTGGAGTTGGGGTTTTCA
Col1a2	CAGAACATCACCTACCACTGC	TTCAACATCGTTGGAACCCTG

Supplementary Table 1: Primer sequences used in qRT-PCR experiments (see Materials and Methods)
